# Recent advances in nano-enabled immunomodulation for enhancing plant resilience against phytopathogens

**DOI:** 10.3389/fpls.2024.1445786

**Published:** 2024-08-07

**Authors:** Hafiza Ayesha Masood, Yetong Qi, Muhammad Khubaib Zahid, Zhitao Li, Salman Ahmad, Ji-Min Lv, Muhammad Shafiq Shahid, Hamada E. Ali, Gabrijel Ondrasek, Xingjiang Qi

**Affiliations:** ^1^ Xianghu Laboratory, Hangzhou, China; ^2^ MEU Research Unit, Middle East University, Amman, Jordan; ^3^ Department of Life Sciences, Western Caspian University, Baku, Azerbaijan; ^4^ Institute of Nuclear Agricultural Sciences, Zhejiang University, Hangzhou, China; ^5^ Department of Plant Pathology, Faculty of Agriculture, University of Sargodha, Sargodha, Pakistan; ^6^ Department of Plant Sciences, College of Agricultural and Marine Sciences, Sultan Qaboos University, Muscat, Oman; ^7^ Department of Biology, College of Science, Sultan Qaboos University, Muscat, Oman; ^8^ Faculty of Agriculture, University of Zagreb, Zagreb, Croatia

**Keywords:** agriculture, nanomaterials, immunomodulation, phytopathogens, disease resistance

## Abstract

Plant diseases caused by microbial pathogens pose a severe threat to global food security. Although genetic modifications can improve plant resistance; however, environmentally sustainable strategies are needed to manage plant diseases. Nano-enabled immunomodulation involves using engineered nanomaterials (ENMs) to modulate the innate immune system of plants and enhance their resilience against pathogens. This emerging approach provides unique opportunities through the ability of ENMs to act as nanocarriers for delivering immunomodulatory agents, nanoprobes for monitoring plant immunity, and nanoparticles (NPs) that directly interact with plant cells to trigger immune responses. Recent studies revealed that the application of ENMs as nanoscale agrochemicals can strengthen plant immunity against biotic stress by enhancing systemic resistance pathways, modulating antioxidant defense systems, activating defense-related genetic pathways and reshaping the plant-associated microbiomes. However, key challenges remain in unraveling the complex mechanisms through which ENMs influence plant molecular networks, assessing their long-term environmental impacts, developing biodegradable formulations, and optimizing targeted delivery methods. This review provides a comprehensive investigation of the latest research on nano-enabled immunomodulation strategies, potential mechanisms of action, and highlights future perspectives to overcome existing challenges for sustainable plant disease management.

## 1 Introduction

Agriculture is the most important sector that plays a crucial role in providing food, supporting economic stability, and maintaining ecological balance worldwide (Hartmann and Six, 2023). Plant pathogens, including fungi, bacteria, viruses, and other pathogens, pose a severe threat to agricultural productivity and food security worldwide (Kim et al., 2023). In the last few decades, due to trade globalization, climate change, and other factors, new pathogens are constantly emerging, and existing diseases are also spreading, posing a serious threat to agricultural production (Singh et al., 2023). Moreover, since the Green Revolution the global population has dramatically raised by more than 5 billion people, and the shortage of typical agricultural methods has critically restrained our capability to conserve food safety (Conway, 1998). Traditional methods such as broad-spectrum antibiotics and chemical pesticides provide some support; however, they also pose adverse effects to the environment and human health and can also lead to drug resistance in plant pathogens (Ahmed et al., 2022). To address these issues, traditional breeding and modern biotechnology are expected to significantly improve plant resilience to various pathogens by improving crop resistance genes and immune signaling pathways (Conway, 1998; Munaweera et al., 2022).

Although genetic modifications of plants offer promising advantages, these require risk assessment and careful consideration to confirm their safety and sustainability (Anders et al., 2021). Therefore, it is important to find new strategies for environmentally friendly and sustainable plant disease management. Enhancing crop resilience to pathogens is one of the key strategies to control plant diseases and maintain agricultural sustainability, while minimizing the reliance on traditional pesticides (Hannan Parker et al., 2022; Ngou et al., 2022). Notably, the innate immune system of plants provides a strong defense against pathogen invasion (Ma et al., 2021), but how to effectively regulate and enhance plant immunity remains to be established. In recent years, the application of nanotechnologies in agriculture has attracted increasing attention, due to their unique nanoscale-specific properties such as high efficiency, large surface area, small size, targeted delivery, and controlled release (Beckers et al., 2021).

Nano-enabled immunomodulation involves the use of engineered nanomaterials (ENMs) as nanoscale agrochemicals to modulate the immune response of plants to improve disease resistance (Ahmed et al., 2024; Zhang et al., 2024). ENMs can be used as nanocarriers to efficiently and precisely deliver immune signaling molecules or genes to specific locations in cells, thereby regulating the plant immune system (Zhang et al., 2021b). Additionally, nanoprobe technology is also used to monitor key biomolecular changes in plant immunity, and biotic and abiotic stress responses in real-time (Son et al., 2023). Recent studies have demonstrated that ENMs application strengthens the plant immunity and tolerance against biotic stress by enhancing systemic acquired resistance (SAR) and induced systemic resistance (ISR), and modulating antioxidative defense systems. For example, the foliar application of silica nanoparticles (SiNPs) 100 mg L^−1^ improved the disease resistance in *Arabidopsis thaliana* plants against bacterial pathogen *Pseudomonas syringae* by inducing the SAR in a dose-dependent manner. SAR-inducing phytohormone such as SA successfully enhances stress tolerance by upregulating the expression of pathogenesis-related genes (El-Shetehy et al., 2021). In another recent study, Noman et al. (2024) reported that salicylic acid (SA) coated biogenic iron nanoparticles (bio-FeNPs) at 100 mg Kg^-1^ concentration significantly suppressed Fusarium wilt disease in watermelon (*Citrullus lanatus* L.) caused by a fungal pathogen *Fusarium oxysporum* f. sp. *niveum* by improving SAR response via triggering antioxidative defense systems and SA signaling pathway genes. These findings suggest that nano-enabled immunomodulation might be an alternative way for enhancing plant resilience against phytopathogens; however, mechanistic insights and translation of these approaches from laboratory to the field scale involves significant challenges.

The aim of this review is to provide a comprehensive overview of the latest research progress in nano-enabled immunomodulation for enhancing plant resilience against phytopathogens threats. We aim to elucidate the potential mechanisms by which ENMs can modulate plant immune responses, critically evaluate the latest research advances, and highlight future challenges and opportunities for translating these approaches into sustainable agricultural practices.

## 2 Dynamics of immunomodulation in plants against phytopathogens

The coevolution of plants and microbial pathogens has led to an intricate interplay of defense and attack mechanisms (Fields and Friman, 2022; Harris and Mou, 2024). Microbial pathogens have developed strategies for evading or suppressing plant immune systems; however, plants employ various sophisticated defense mechanisms in response (Sun and Zhang, 2021). Notably, effector-triggered immunity (ETI) and pattern-triggered immunity (PTI) are two primary defense mechanisms of innate plant immunity against phytopathogens. In plants, NOD-like receptors (NLRs) constitute sensor and helper NLRs, which are responsible for ETI (Laflamme et al., 2020; Nabi et al., 2024). For example, Wang et al. (2023) reported that *MPK3* and *MPK6* suppression in Arabidopsis can potentially reduce pre-PTI-mediated ETI suppression (PES) through inhibition of two protein phosphatases genes (*AP2C1* and *PP2C5*). Furthermore, recognition of conserved microbial features such as microbe-associated molecular patterns (MAMPs) through plant pattern recognition receptors (PRRs) can initiate PTI. However, phytopathogens can evade PTI by shielding or modifying MAMPs, inhibition of PRRs or downstream signaling components by secreting effectors (Lü et al., 2022; Loo et al., 2022; Totsline et al., 2023). Lee et al. (2024) reported that AVRblb2 pathogen effector forms a complex with calmodulin-like (CML) and calmodulin (CaM) proteins to interact with NbCNGC18 to disrupt PAMP-triggered immunity signaling. These mechanisms can potentially hijack plant defense systems, driven by the coevolutionary relationship between plants and phytopathogens (Sood et al., 2021; Harris and Mou, 2024).

**Figure 1:**
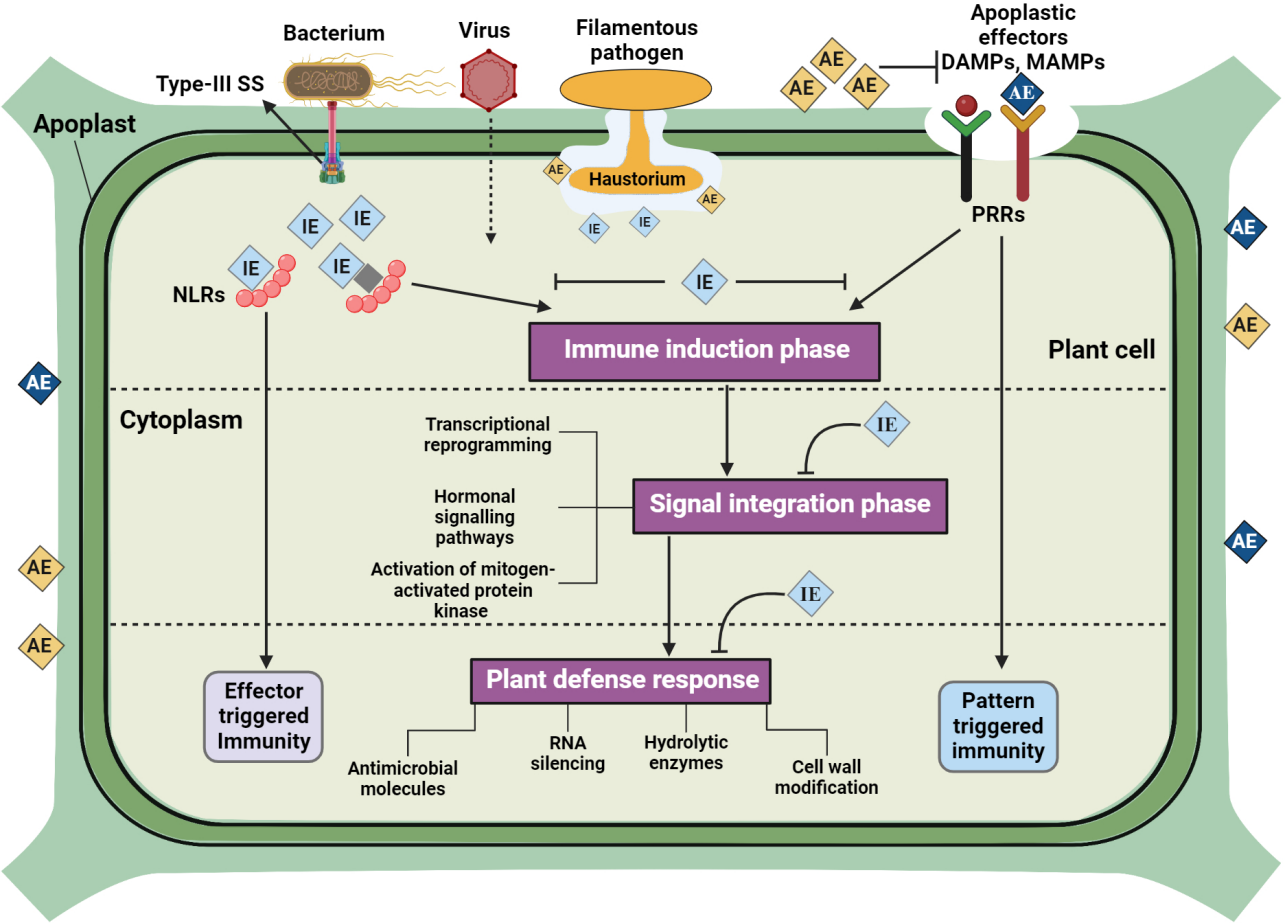
Schematic representation of plant immunomodulation against microbial pathogens. Microbial pathogens invasion initiates plant defense responses in three phases including immune recognition, signal integration, and defense response. Firstly, plants perceive pathogen-associated molecular patterns (PAMPs), damage-associated molecular patterns (DAMPs), and microbial effectors through intracellular and surface receptors. Secondly, various immune signaling events are activated, which involve the integration of immune signals from the recognition of diverse patterns and effectors. Finally, an effective and swift defense response is initiated in each cellular compartment of the plant cell, which leads to pattern-triggered immunity (PTI) and NLR-mediated pathways triggering the effector-triggered immunity (ETI) response. Host resistance is modulated by the cumulative action of effectors transmitted by microbial pathogens.

Pathogen effectors can also play a central role in modulating plant immunity (Dou and Zhou, 2012), which target various components of plant immune system, from early recognition events to downstream signaling and defense responses (**Figure 1**). Effectors can prevent release, binding, or perception of MAMPs, or inhibit key signaling hubs such as receptor-like cytoplasmic kinases, interfere with PRR complexes, MAPK cascades, and phytohormone signaling pathways (Ceulemans et al., 2021; Iswanto et al., 2022). In a recent study, Qiu et al. (2023b) reported that inhibition of *GmLHP1-2*/*GmPHD6* complex transcriptional activity in soybean due to suppressing effect of PsAvh110 nuclear effector from *Phytophthora sojae* can potentially evade plant immunity response. In another study, Shang et al. (2024) reported that CfEC12 (a fungal effector) from *Colletotrichum fructicola binds to* MdNIMIN2 and disrupting its interaction with MdNPR1 leading to suppression of salicylic acid defense pathway.

Pathogens create a favorable environment for infection by rewiring plant immune signaling networks. In addition to suppressing immune signaling pathways, pathogens also target and manipulate the downstream defense outputs of the plant immune system (Mishra et al., 2021; Singh et al., 2023). Effectors and secreted enzymes help pathogens overcome physical and chemical barriers in the plant, such as cell wall reinforcements, antimicrobial compounds, and hydrolytic enzymes. Some pathogens produce toxins or phytohormone mimics that further manipulate plant physiology and development to their advantage (Kaur et al., 2022; Wang et al., 2022). The dynamics of plant-pathogen interactions are further shaped by the spatiotemporal regulation of immunity and infection processes (König et al., 2021). Plants must balance the allocation of resources between growth and defense, while pathogens face the challenge of avoiding detection and preserving host viability. Therefore, timing and localization of immune responses and pathogen colonization are critical determinants of disease outcomes (Li et al., 2020; Monson et al., 2022). Taken together, the dynamics of plant immunomodulation against phytopathogens involve an intricate interplay of recognition mechanisms that detect PAMPs, DAMPs and effector molecules, subsequent signaling cascades that transmit this detection, and the activation of diverse defense responses. Furthermore, the application of ENMs to modulate plant immune responses has emerged as a promising alternative strategy for enhancing plant disease resistance and management (Liu et al., 2024; Zhang et al., 2024).

## 3 Nano-enabled technologies for plant immunomodulation

**Figure 2:**
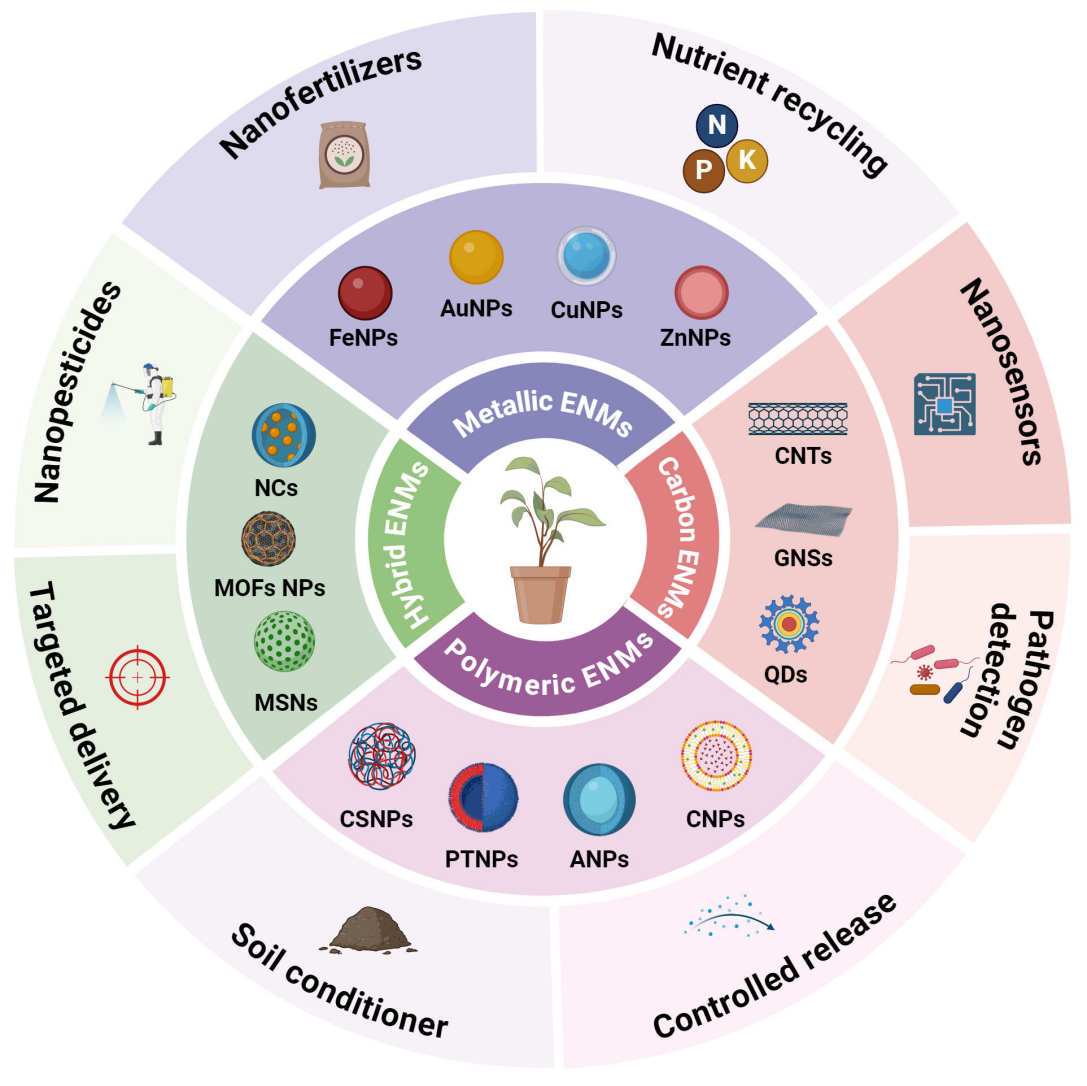
Schematic illustration of the current engineered nanomaterials (ENMs) toolbox to improve plant immunity against microbial pathogens. ENMs can be divided into different groups based on their unique physiochemical properties. nanoparticles (NPs), metal-organic framework (MOFs), nanocapsules (NCs), mesoporous silica nanoparticles (MSNs) chitosan (Cs), pectin (Pt), cellulose (Cl), alginate (A), quantum dots (QDs), graphene nanosheets (GNSs), carbon nanotubes (CNs), iron (Fe), copper (Cu), gold (Au) and zinc (Zn).

The plant immune system is essential for maintaining plant health and responding to pathogen invasions. However, the traditional methods (conventional breeding, crop rotation chemical fertilizers and pesticides) for activating or regulating plant immunity have shortcomings such as low efficiency and poor targeting (Raymaekers et al., 2020; Ma et al., 2021). Nano-enabled agriculture provides a new idea for the precise regulation of plant immunity and reducing the dependence on chemical pesticides (Fiol et al., 2021). Nano-enabled immunomodulation mechanisms lies in the ability of ENMs to act as nanoscale delivery platform for immunomodulatory agents (Noman et al., 2023b). Notably, ENMs as nanoscale carrier can efficiently carry immune signaling molecules (such as protein, enzymes, hormones, and RNA, etc.) into plant cells and activate immune responses (Vega-Vásquez et al., 2021; Liu et al., 2024). ENMs can be divided into different groups (metal based or inorganic ENMs, carbonaceous ENMs, polymer ENMs and hybrid ENMs) based on their unique nanoscale properties including size, shape, crystalline structure, chemical composition (**Figure 2**) (Saleh, 2020). Metal-based or inorganic ENMs mainly including zinc (Zn), gold (Au), silver (Ag), copper (Cu), titanium (Ti) and silica, which shown great potential for enhancing plant immunity against pathogens and environmental stresses (Mitchell et al., 2021). These inorganic ENMs can be produced to desired properties, geometries, sizes, and with desired functionalization/coatings to optimize benefit. Ma et al. (2020) reported that nanoscale Cu (250 mg L^−1^) amendments significantly suppressed soybean sudden death syndrome by activating plant immunity and enhancing the phytohormone contents, photosynthetic endpoints, antioxidant enzymes and nutritional status.

Carbon-based ENMs including carbon dots (CDs), carbon nanotubes (CNTs) and graphene NPs have been shown to promote plant growth and resilience against pathogens (Li et al., 2023). For example, Adeel et al. (2021) demonstrated that foliar exposure of CNTs at 200 mg L^−1^ significantly suppressed tobacco mosaic virus infection by activating of the defense system in tobacco (*Nicotiana benthamiana*) plants. Additionally, CNTs application enhanced the plant immunity by triggering defense-related phytohormones, antioxidant enzymes and improving photosynthetic performance. Polymeric ENMs such as polylactic acid, chitosan, pectin, carboxymethyl cellulose and alginate have attracted recent attention for controlled and sustained release of amicrobial agents and protect plants against pathogens (Shakiba et al., 2020; Vemula and Reddy, 2023). In a recent study, Hafeez et al. (2024) reveled that biologically produced chitosan NPs enhanced the rice blast disease resistance by improving antioxidant defense system (SOD, APX and CAT), nutrient uptake, photosynthesis efficiency and reducing the cellular oxidative stress (MDA and H_2_O_2_) in rice (*Oryza sativa* L.) plants. Nanohybrid, such as liposomes, nanocapsules (NCs), nanoemulsions and mesoporous silica nanoparticles (MSNs) can be engineered to carry pesticides, nutrients, enzymes and phytohormones for targeted delivery. Abdelrasoul et al. (2020) demonstrated that monoterpenes-based nanoemulsions at 100 mg L^-1^ concentration inhibited the *Pectobacterium carotovorum* and *Ralstonia solanacearum* pathogens growth and induced systemic resistance in potato (*Solanum tuberosum* L.) leaves by improving antioxidant enzymes activity. Overall, ENMs are able to modulate plant immune responses through multiple pathways, not only enhancing plant resistance to pathogens, but also promoting crop growth and increasing yields (**Figure 2**). However, there is still little understanding of the translocation, transformation, residue, and long-term environmental impact of ENMs in plants, and further research needs to be explored the potential mechanisms. In addition, the development of more environmentally friendly, efficient, and biodegradable ENMs formulations is also a key direction in future research.

## 4 Mechanisms of nano-enabled immunomodulation in plants

### 4.1 Nano-enabled activation of phytohormone signaling

**Figure 3:**
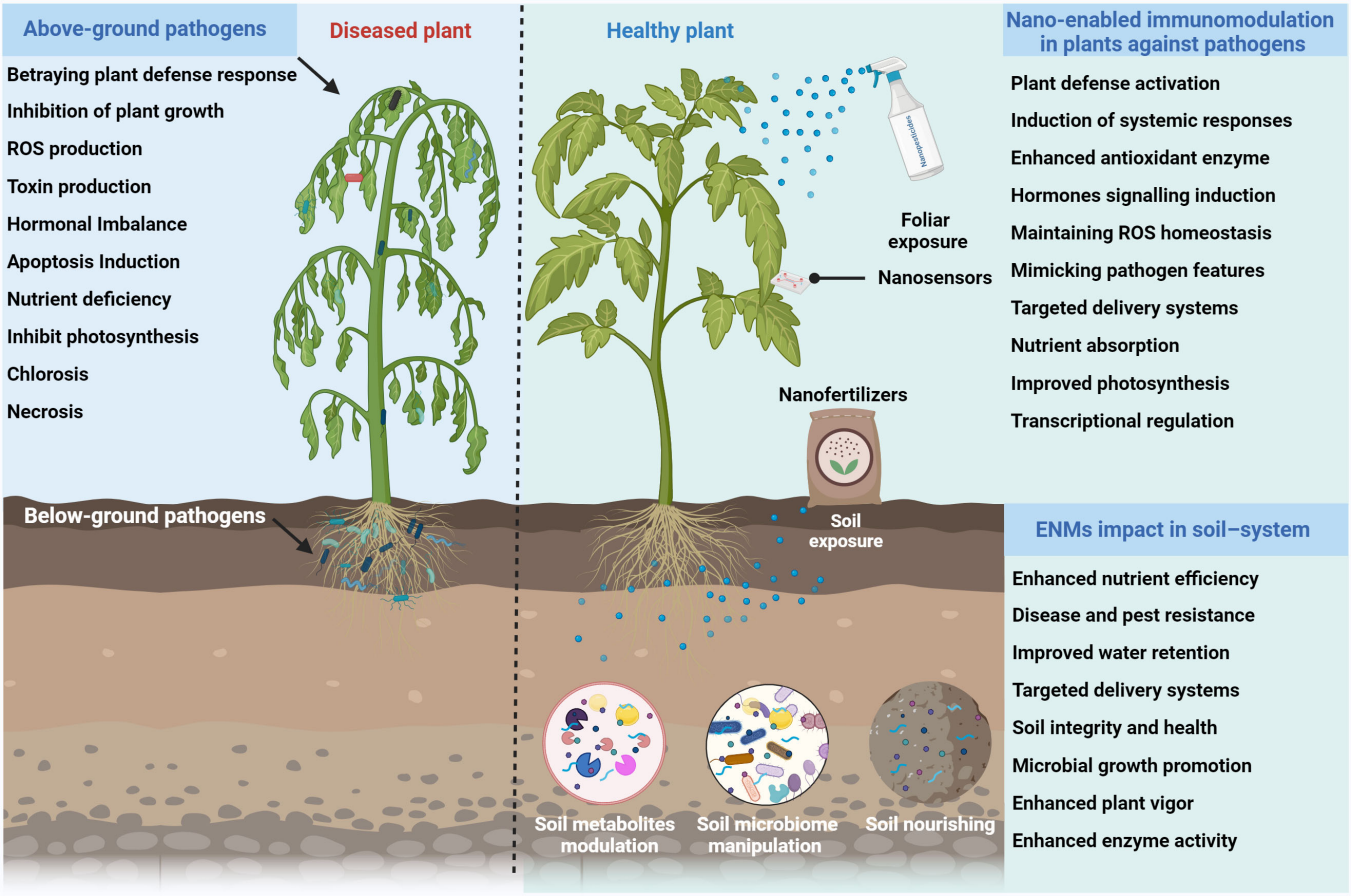
Schematic representation of nano-enabled immunomodulation to revolutionize plant health through several mechanisms. On the left, the diseased plant shows symptoms due to various pathogen-induced mechanisms, which inhibit plant growth. On the right, foliar and soil exposure to ENMs enhance plant growth by activating systemic responses, enhancing antioxidant enzyme activity, inducing hormone signaling, and maintaining ROS homeostasis.

**Figure 4:**
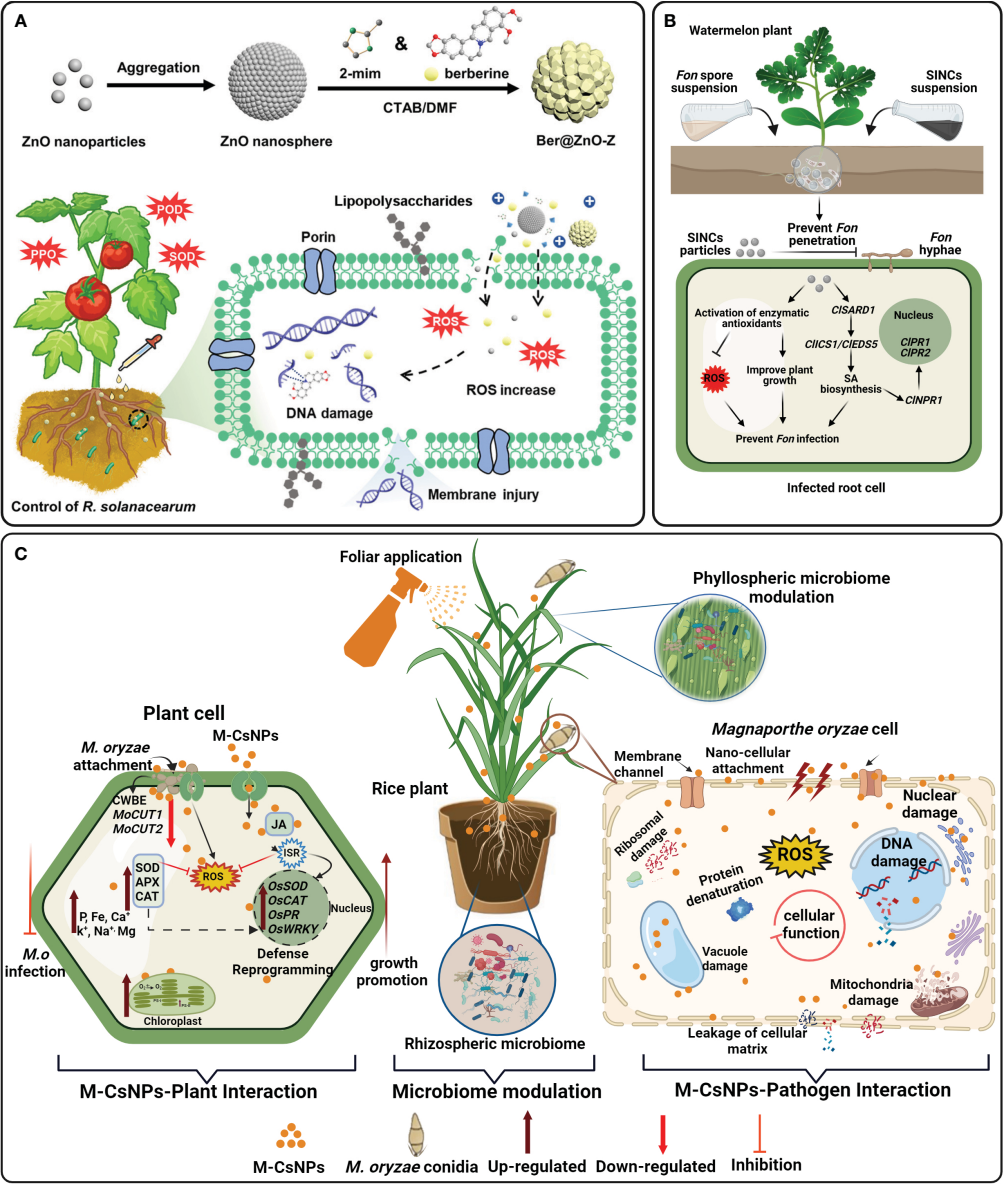
Examples of engineered nanomaterials (ENMs) used for plant disease management through immunomodulation. **(A)** The figure illustrates the synthesis of berberine-loaded ZnO-Z (Ber@ZnO-Z) nanosphere to synergistically control the bacterial wilt disease in tomato plants through direct pathogen inhibition and modulating antioxidant enzymes. Adapted with permission from reference (Liang et al., 2022). Copyright 2022 American Chemical Society. **(B)** Therapeutic delivery of salicylic acid-coated biogenic iron nanocomposites (BINCs) suppressed Fusarium wilt damages in watermelon plants by inducing systemic acquired resistance (SAR) and modulating antioxidative defense system. Adapted with permission from reference (Noman et al., 2024). Copyright 2024 Elsevier. **(C)** Schematic representation of the proposed mechanism of moringa chitosan nanoparticles (M-CsNPs) to control bacterial rice blast disease of rice. M-CsNPs act as nanofungicides that inhibit the pathogen *Magnaporthe oryzae* infection and improve defense responses by improving biochemical attributes and regulating transcriptional traits and modulating plant-associated microbiome. Adapted with permission from reference (Hafeez et al., 2024). Copyright 2024 Elsevier.

Phytohormone signaling pathways are involved in inducing a variety of defense responses against biotic and abiotic stresses. Plant hormones such as jasmonic acid (JA), salicylic acid (SA), ethylene (ET) and abscisic acid (ABA) play an important role in the plant immune response against pathogens (Huang et al., 2020; Zhao et al., 2021). In recent years, nano-enabled activation of plant hormone signaling represents an innovative approach in plant disease management (**Figure 3**). ENMs can design too slowly release hormones in a controlled manner, thus activating signaling pathways and enhancing the plant resistance to microbial pathogens (Tripathi et al., 2022; Liu et al., 2024). For example, Noman et al. (2024) observed that soil-application with SA-doped FeNPs suppressed the Fusarium wilt disease in watermelon through inhibiting the fungal invasive growth and improving the antioxidative capacity, and primed a SAR response via activating the SA signaling genes (**Figure 4B**). In another study, lanthanum oxide NMs at 200 mg L^-1^ with different surface modifications significantly suppressed cucumber wilt disease by 12.50–52.11% by improving total amino acids, vitamin contents, and activating SA-dependent systemic acquired resistance (Cao et al., 2023a). Similarly, elemental sulfur NPs increase resistance against Fusarium wilt disease, caused by a fungal pathogen *Fusarium oxysporum* f. sp. *lycopersici*. Notably, sulfur NPs 30–100 mg L^-1^ suppressed pathogen infection by regulating the SA-dependent systemic acquired resistance pathway and modulating of the expression of antioxidase-related and pathogenesis-related genes.

Polymeric ENMs have also demonstrated excellent ability to regulate plant hormone signaling (**Table 1**). For example, Giri et al. (2023) reported that chitosan fabricated AgNPs control the bacterial leaf spot disease in tomato plants by inducing SAR mechanism through upregulating stress hormones responsive genes (*PR1*, *NHO1*, *NPR1*, *MYC2*, *JAR1*, *ERF1*). Taken together, these previous studies confirm that ENMs can precisely regulate plant immune responses by influencing hormone concentrations and regulating signaling pathways. However, the mechanism of action of different ENMs is different, and the specific effect may also vary depending on the plant species and the type of pathogen. In the future, it is necessary to study the interaction between ENMs and molecular networks in plants and explore new strategies to improve the specificity of immune mechanisms.

**Table 1:** Potential applications of engineered nanomaterials (ENMs) for improving immunomodulatory mechanisms and enhancing disease resilience in agricultural crops.

ENMs	ENMs concentrations	Target pathogens	Host plants	Mechanisms	References
Chitosan NPs	200 mg L^−1^	*Magnaporthe oryzae*	Rice	Enhanced disease resistance by improving antioxidant enzymes, photosynthesis pigments and reshaping the rhizosphere microbiome	(Hafeez et al., 2024)
Chitosan-coated silica NPs	200 mg L^−1^	*Fusarium virguliforme*	Soybeans	Reduced disease incidence by improving chlorophyll and micronutrient contents	(O’Keefe et al., 2024).
Chitosan coated zinc oxide NPs	200 µg mL^−1^	*Pseudomonas syringae* pv. *tomato*	Tomato	Suppressed bacterial speck disease by improving photosynthesis parameters	(Esserti et al., 2024)
Chitosan coated iron NPs	250 mg L^−1^	*Xanthomonas oryzae* pv. *oryzae*	Rice	Reduced disease incidence by inducing plant antioxidative defense mechanisms, and modulating microbiome	(Ahmed et al., 2022)
Copper chitosan NPs	0.16% w/v	*Curvularia lunata*	Maize	Enhanced defense responses by regulating antioxidant enzymes activity and photosynthesis profile	(Choudhary et al., 2017)
Copper oxide NPs	10-50 ppm	*Fusarium oxysporum*. f. sp. *ciceris*	Chickpea	Enhanced disease resistance by increasing photosynthetic rate, protein, tannin, phenolics, and flavonoid and enzyme contents	(Tiwari et al., 2024)
Copper NPs	31.25 mg L^–1^	*Rhizoctonia solani*	Tomato	Suppressed disease progression by activating antioxidative defense response and improving chlorophyll contents	(Shen et al., 2020)
Copper NPs	100 µg mL^−1^	*Acidovorax citrulli*	Watermelon	Activated antioxidant enzymes and stomatal immunity for disease suppression	(Noman et al., 2023c)
Zinc oxide NPs	–	*Fusarium oxysporum*	Tomato	Enhanced disease resistance by inducing plant defense responses	(Bouqellah et al., 2024)
Zinc oxide NPs	500 µg mL^−1^	*Fusarium oxysporum*	Eggplant	Reduced disease incidence by activating plant biochemical defense mechanisms	(Abdelaziz et al., 2022)
Sulfur NPs	200 mg L^−1^	*Fusarium oxysporum* f. sp. *Lycopersici*	Tomato	Preserved the enrichment of plant beneficial bacteria	(Steven et al., 2024).
Sulfur NPs	100 mg L^−1^	*Fusarium oxysporum* f. sp. *Lycopersici*	Tomato	Reduced disease incidence by activating SA-mediated disease resistance mechanisms	(Cao et al., 2021)
Sulfur NPs	100 mg L^−1^	*Pectobacterium carotovorum*	Lettuce	Decreased the disease occurrence by activating SA- and JA-dependent pathways	(Cao et al., 2023b)
Lanthanum silicate NPs	100 mg L^−1^	*Rhizoctonia solani*	Rice	Enhanced disease resistance by regulating SAR immune responses	(Cao et al., 2023a)
Selenium	5 mg L^−1^	*Rhizoctonia solani*	Rice	Suppressed disease by promoting flavonoid biosynthesis, antioxidative system and SA -dependent acquired disease resistance	(Chen et al., 2024)
SA-coated iron NPs	100 mg Kg L^−1^	*Fusarium oxysporum* f. sp. *niveum*	Watermelon	Suppressed Fusarium wilt by inducing SAR response via activating antioxidative capacity and SA signaling pathway	(Noman et al., 2024)
Iron NPs	0.25 mM	*Fusarium oxysporum*	Cucumber	Reduced the disease incidence by improving morphological traits and photosynthetic pigments	(El-Batal et al., 2023)
Silver NPs	20 ppm	*Alternaria solani*	Tomato	Inhibited disease incidence by activating antioxidant enzymes and maintaining ROS homeostasis	(Narware et al., 2024)
Silver NPs	100 mg L^−1^	*Xanthomonas oryzae* pv. *oryzae*	Rice	Decreased disease occurrence by regulating plant antioxidative defense system	(Ahmed et al., 2020)
Manganese NPs	100 µg mL^−1^	*Fusarium oxysporum* f. sp. *niveum*	Watermelon	Suppressed disease progression by activating antioxidative defense response, phytohormones and modulating microbial community	(Noman et al., 2023a)
Manganese NPs	500 µg mL^−1^	*Fusarium oxysporum* f. sp. *niveum*	Watermelon	Decreased disease occurrence by regulating the expression of defense-related genes	(Elmer et al., 2018)
Titanium dioxide NPs		*Ralstonia solanacearum*	Tomato	Increased disease resistance by regulating antioxidative immune responses	(Pan et al., 2023)
Quantum dots	50 mg L^−1^	*Verticillium dahliae*	Cotton	Suppressed disease by maintaining ROS homeostasis	(Qiu et al., 2023a)
Silica NPs	25-1600 mg L^−1^	*Pseudomonas syringae*	*Arabidopsis thaliana*	Increased disease resistance by inducing SAR immune responses	(El-Shetehy et al., 2021)
MOFs NPs	5-15 mg L^−1^	*Phytophthora infestans*	Wheat	Controlled release of fungicide significantly inhibited the fungal pathogen and improved the plant growth	(Shan et al., 2020)
MOFs NPs	1 mg L^−1^	*Rhizoctonia solani*	Rice	*In vitro* studies showed the antifungal activity of MOFs, while also enhancing plant growth	Huang et al., 2023)

### 4.2 Nano-enabled stimulation of antioxidant defense system

Nano-enabled stimulation of the plant antioxidant defense system offers a promising approach to mitigating the deleterious effects of oxidative stress induced by pathogens (Pan et al., 2023). This system is designed to neutralize reactive oxygen species (ROS) generated during pathogen attacks or other stress conditions. ROS can cause oxidative damage to cellular components, including lipids, proteins, and DNA, ultimately leading to cell death and tissue dysfunction (Chen et al., 2023). The antioxidant defense enzymes (CAT, SOD, POD, APX, PPO) acts as a frontline defense, mitigating the detrimental effects of ROS and protecting plant cells from oxidative stress (Dvořák et al., 2021). ENMs with their unique physicochemical properties, have demonstrated the ability to modulate the plant antioxidant defense system. Notably, ENMs can interact with plant cells and trigger specific signaling pathways (**Figure 3**), leading to the upregulation of antioxidant enzymes and the biosynthesis of non-enzymatic antioxidants (Abdelrhim et al., 2021; Tiwari et al., 2024). For example, Noman et al. (2023c) investigated that foliar exposure of biogenic CuNPs at 100 µg mL^−1^ concentration substantially suppressed bacterial fruit blotch disease in watermelon plants by triggering antioxidants enzymes (CAT, SOD and POD), modulating stomatal immunity, and reducing the ROS activity.

In another study, biologically synthesized AgNPs improved early blight disease resistance by enhancing antioxidant enzymes (CAT, LPX, PO, SOD), and maintaining ROS (H_2_O_2_ and O^−^_2_) homeostasis in tomato plants (Narware et al., 2024). Similarly, FeO nanocomposites control the cucumber wilt disease caused by *Fusarium oxysporum* by stimulating morphological performances, total phenol, soluble protein contents, photosynthetic pigments and antioxidant enzymes (POD and PPO) (El-Batal et al., 2023). Liang et al. (2022) demonstrated that berberine loaded ZnO NMs at 100-1000 ug mL^-1^ significantly reduced the tomato bacterial wilt disease severity by 45.8% by improving the plant growth and antioxidant enzymes (SOD, PPO, PPO) (**Figure 4A**). Previous studies primarily focused on the mitigation of pathogen-induced oxidative stress by enhancing antioxidant activity (**Table 1**). However, ongoing research exploring biocompatible ENMs formulations and targeted delivery methods holds immense potential for sustainable agriculture and food security.

### 4.3 Nano-enabled regulation of photosynthesis and nutritional profile

Photosynthesis is an important process that drives plant growth and development, is often disrupted by pathogen infections (Yang and Luo, 2021). Phytopathogens can negatively impact photosynthesis by disrupting the structure and function of photosynthetic apparatus, reducing the efficiency of light harvesting, and impairing carbon fixation. Consequently, this can lead to reduced plant productivity, compromised nutrient acquisition, and an overall decline in plant health (Parveen and Siddiqui, 2021; Karpagam et al., 2023). In recent years, ENMs mediated regulation of photosynthesis and the nutritional profile of plants represents a promising strategy in sustainable agriculture, offering a multifaceted approach to mitigating the impacts of pathogens on crop productivity and quality (**Figure 3**). Importantly, ENMs can interact with plant cells and trigger specific signaling pathways, leading to the upregulation of photosynthetic enzymes, the biosynthesis of pigments, and the modulation of nutrient uptake and assimilation (Ahmed et al., 2022; Parveen and Siddiqui, 2022). For example, the foliar application of ZnONPs at 0.20 mL^−1^ concentrations significantly enhanced the plant growth and photosynthesis efficiency (Total chlorophyll and carotenoids contents) of tomato plants under several bacterial and fungal pathogens infection (Parveen and Siddiqui, 2021).

In another recent study, Esserti et al. (2024) chitosan embedded ZnONPs effectively control the bacterial speck disease of tomato by improving the plant growth, photosynthetic pigments (Chlorophyll a, Chlorophyll b, carotenoids), and gas exchange parameters such as internal CO_2_ concentration, net photosynthesis rate, transpiration rate and stomatal conductance. Notable, this study showed that bacterial speck disease significantly affected plant biomass and photosynthetic performance; however, NP applications mitigate the negative impact of phytopathogens. Likewise, Mogazy et al. (2022) reported that Ca and FeNPs at (100 and 200 ppm) positively regulate innate immune responses in strawberry (*Fragaria ananassa*) plants against gray mold disease caused by a fungal pathogen *Botrytis cinerea*. This study revealed that foliar exposure of NPs significantly increased the vitamin, phenolics, and flavonoids contents, and nutritional profile (Zn^2+^, Mg^2+^, Ca^2+^, Fe, N, P, and K^+^) in strawberry plants as compared to infected control. Likewise, chitosan coated mesoporous SiNPs treatment significantly reduced the sudden death syndrome by 30% and increased the micronutrient (Zn, Mn, Mg, K, B) content, and chlorophyll efficiency in soybean plants (O’Keefe et al., 2024). Taken together, previous studies demonstrated that nano-enabled regulation of nutritional profiles and photosynthesis shows great promise in mitigating pathogen impacts on crops (**Table 1**). However further research on ENMs toxicity, application methods, and biosafety is recommended for sustainable agricultural applications.

### 4.4 Nano-enabled modulation of microbiome and metabolites

The plant-associated microbiome (bacteria, fungi, viruses) plays a crucial role in plant health, growth, and resistance against pathogens (Trivedi et al., 2020; Steven et al., 2024). The plant-associated microbiome serves as a frontline defense against pathogens, conferring protection through various mechanisms, such as competitive exclusion, antimicrobial compound production, and the induction of systemic resistance pathways in plants (Fitzpatrick et al., 2020; Wang et al., 2022). Additionally, the intricate network of metabolites produced by plants and their associated microbiomes acts as a defensive arsenal against invading pathogens (Liu et al., 2020; Rangel and Bolton, 2022). However, microbial pathogens can disrupt the delicate balance of the plant microbiome, leading to dysbiosis and compromising plant health (Zhang et al., 2021a). Nano-enabled microbiome engineering has recently emerged as a powerful platform to enhance plant resilience against pathogenic threats (Ahmed et al., 2023; Hussain et al., 2023). In recent years, several studies have demonstrated the potential impact of ENMs plant-associated microbiome under biotic stress condition. The application of biogenic chitosan-Fe nanocomposite (BNCs) at 250 μg mL^−1^ concentration significantly reduced the bacterial leaf blight (BLB) disease incidence (67.1%) by enhancing the relative abundance of beneficial bacterial community such as *Allorhizobium*, *Ochrobactrum*, *Pseudolabrys*, *Sphingomonas*, *Devosia*, *Bradyrhizobium* and *Methylobacterium* in rice plants (Ahmed et al., 2022).

Additionally, BNCs amendments also enhanced the rice plant growth by modulating antioxidant enzymes, enhancing photosynthesis efficiency, and reducing ROS activity. Similarly, Noman et al. (2023a) revealed that soil application of biologically synthesized manganese (Mn) NPs control the Fusarium wilt disease in watermelon by enhancing SAR mechanism via triggering antioxidative defense machinery, SA signaling pathway, and modulating the soil bacterial community (*Sphingomonas*, *Gemmatimonadaceae*, *Nocardioides*, and *Burkholderiaceae*) and fungal community (*Penicillium*, *Botryotrichum*, *Conocybe*, and *Mortierella*). The foliar spray of nitrogen-doped CDs (10 mg L^−1^) alleviated tomato bacterial wilt disease induced damage by 71.2% through indirect resistance activation (SAR activation) and ROS scavenging. Moreover, metabolomics profile revealed that nitrogen doped CDs significantly improved the fatty acid and tricarboxylic acid synthesis in tomato plants (Luo et al., 2021). Similarly, the application of sulfur NMs at 10–100 mg L^-1^ significantly decreased the occurrence of bacterial soft rot disease in lettuce (*Lactuca sativa* L.) plants by improving the chlorophyll contents, antioxidant enzymes and regulating the defense-related genes expression. In addition, metabolomics analysis showed that sulfur NMs enhanced the tricarboxylic acid cycle and also regulated SA and JA metabolite biosynthesis, thereby enhancing the bacterial soft rot disease resistance in lettuce (Cao et al., 2023b). Taken together, nano-enabled modulation of microbiome and metabolites profile to enhance plant disease resistance has the potential to serve as highly sustainable, efficient, sustainable, and non-toxic alternative for the management of plant diseases (**Figure 3**).

### 4.5 Nano-enabled activation of defense related genetic pathways

Plants have evolved intricate defense mechanisms to protect themselves against a wide range of microbial pathogens. These defense responses are governed by complex genetic pathways that involve the coordinated expression of numerous genes encoding various proteins, enzymes, and signaling molecules (Kaur et al., 2022). After pathogens attack, specific defense-related genes are activated, triggering a cascade of events that ultimately lead to the production of antimicrobial compounds, the reinforcement of physical barriers, and the activation of systemic resistance pathways (Nishad et al., 2020; Dodds et al., 2024). Nano-enabled activation of defense-related genetic pathways represents a promising strategy in sustainable agriculture, offering a targeted and efficient approach to enhancing plant resilience against pathogenic threats (Cao et al., 2023a). For example, selenium (Se) NMs application 5 mg L^-1^ decreased the disease severity (68.8%) by enhancing the organic Se content (44.8%), nutritional quality by (7.2%) and rice yield up to (31.1%). Additionally, metabolomic and transcriptomic analyses confirmed that SeNMs simultaneously boosted the SA and JA dependent acquired disease resistance pathways, flavonoid biosynthesis and antioxidative defense system. Notably, Importantly, SeNMs significantly upregulated the expression of genes *LOX2*, *LOX3*, *LOX6*, *OPR1*, *PR1*, *PR3*, *AOC*, and *JAR* while reducing the expression level of genes *POD*, *CAT*, and *SOD2* in rice plants compared to the infected controls, indicating overall stimulation of SAR in SeNM-treated rice (Chen et al., 2024).

Similarly, Hafeez et al. (2024) revealed that foliar exposure of biogenic chitosan NPs at 200 mg L^−1^ significantly control the rice blast disease by triggering defense related genes expression such as (*OsNPP1*, *OsGRF9, WRKY71, OsAPX, OsSOD, OsCAT, OsNPR1, OsPR1, OsPR9*, and *MoCUT2*) in rice plants (**Figure 4C**). In another study, seed primed with AgNPs enhanced the rice blast disease resistant by triggering transcriptional and metabolic reprogramming in rice seeds. In this study, KEGG pathway of transcriptomics data demonstrated that AgNPs-priming activated stress signaling and defense related pathways, such as MAPK signaling pathway, flavonol biosynthesis, glutathione metabolism, plant hormone signal transduction, and plant−pathogen interaction (Yan et al., 2022). Similarly, the application of ROS-generating AgNPs as nano-stimulants significantly triggered plant immune/stress responses against rice blast disease. The disease resilience mechanisms showed that AgNPs mediated “stress memory” induced considerable transcriptional reprogramming in rice leaves by modulating the expression of defense genes, including pathogen-plant interaction genes, cell membrane lipid metabolism genes, specialized metabolite biosynthesis-related genes, and other genes related to biosynthesis. These studies have demonstrated the potential of nanotechnology-mediated activation of defense-related gene expression in enhancing crop resilience against pathogens. However, further research is needed to explore the molecular signaling pathways involved in the interactions between ENMs and plant pathogens, as well as their co-stimulating impact on plant defense against phytopathogens.

## 5 Concluding remarks and future outlook

The promising field of nano-enabled immunomodulation has demonstrated exceptional opportunities to enhance plant resilience against a multitude of phytopathogenic threats (Zhang et al., 2024). The unique physicochemical properties of ENMs, coupled with their ability to interact with and modulate intricate plant defense mechanisms, have positioned them as promising tools in sustainable disease management strategies (Singh et al., 2024). However, as this domain continues to evolve, several critical considerations and future research directions must be addressed to harness the full potential of these innovative approaches. Firstly, while significant progress has been made in elucidating the underlying mechanisms through which ENMs influence plant immune responses, a comprehensive understanding of the complex interplay between ENMs and the intricate molecular networks governing plant defense remains elusive (Zhang et al., 2020; Kumar et al., 2024). Future endeavors should focus on unraveling the intricate signaling cascades, transcriptional regulation by ENMs, enabling the development of more targeted and efficient immunomodulatory strategies (Ma et al., 2023b; Zhang et al., 2024).

Additionally, the transformation of nano-enabled immunomodulation from laboratory-scale studies to field applications necessitates rigorous investigations into the environmental fate, behavior, and potential risks associated with the use of ENMs in agricultural settings (Ahmed et al., 2023; Ma et al., 2023a). Comprehensive assessments of the long-term impacts on soil health, nutrient cycling, and ecosystem dynamics are imperative to ensure the responsible and sustainable integration of these technologies into agricultural practices (Ijaz et al., 2023). Furthermore, the development of environmentally friendly, biodegradable, and biocompatible ENM formulations should be a priority, minimizing potential adverse effects on non-target organisms and ensuring compatibility with diverse plant species and environmental conditions (Balusamy et al., 2023; Wahab et al., 2024). Interdisciplinary collaborations between material scientists, plant biologists, and ecotoxicologists could facilitate the design and synthesis of tailored nanomaterials that balance efficacy, sustainability, and biosafety considerations (Shelar et al., 2023; Zain et al., 2023).

Another crucial aspect that warrants attention is the optimization of ENM delivery methods and application techniques. Developing efficient and targeted delivery systems, such as nanocarriers or nanoemulsions, could enhance the bioavailability and site-specific delivery of immunomodulatory agents, minimizing potential off-target effects and maximizing the desired immune responses (Ma et al., 2023c; Jeon et al., 2024). By addressing these critical considerations and leveraging the transformative potential of nanotechnology, researchers and agricultural stakeholders can revolutionize plant disease management practices, contributing to a more resilient, sustainable, and secure global food system.

## Author contributions

HM: Investigation, Resources, Software, Validation, Visualization, Writing – original draft. YQ: Data curation, Investigation, Resources, Software, Visualization, Writing – original draft, Writing – review & editing. MZ: Conceptualization, Resources, Software, Validation, Visualization, Writing – original draft, Writing – review & editing. ZL: Formal analysis, Funding acquisition, Investigation, Software, Supervision, Validation, Writing – review & editing. SA: Conceptualization, Data curation, Investigation, Validation, Writing – review & editing. J-ML: Data curation, Formal analysis, Investigation, Software, Validation, Writing – review & editing. MS: Data curation, Formal analysis, Investigation, Validation, Writing – review & editing. HA: Conceptualization, Resources, Validation, Visualization, Writing – review & editing. GO: Data curation, Funding acquisition, Software, Validation, Visualization, Writing – review & editing. XQ: Conceptualization, Data curation, Funding acquisition, Investigation, Software, Supervision, Visualization, Writing – original draft, Writing – review & editing.
